# Post-exposure immunotherapy for two ebolaviruses and Marburg virus in nonhuman primates

**DOI:** 10.1038/s41467-018-08040-w

**Published:** 2019-01-10

**Authors:** Jennifer M. Brannan, Shihua He, Katie A. Howell, Laura I. Prugar, Wenjun Zhu, Hong Vu, Sergey Shulenin, Shweta Kailasan, Henna Raina, Gary Wong, Md Niaz Rahim, Logan Banadyga, Kevin Tierney, Xuelian Zhao, Yuxing Li, Frederick W. Holtsberg, John M. Dye, Xiangguo Qiu, M. Javad Aman

**Affiliations:** 1 US Army Medical Research Institute of Infectious Diseases, 1425 Porter St, Frederick, MD 21702 USA; 2 Special Pathogens Program, National Microbiology Laboratory, Public Health Agency of Canada, 1015 Arlington Street, Winnipeg, MB R3E 3R2 Canada; 3 Integrated BioTherapeutics, Inc., 4 Research Court, Suite 300, Rockville, MD 20850 USA; 4 Department of Medical Microbiology, University of Manitoba, 745 Bannatyne Avenue, Winnipeg, MB R3E 0J9 Canada; 5 Department of Microbiology and Immunology, University of Maryland School of Medicine, 685 West Baltimore Street, Baltimore, MD 21201 USA; 6 Institute for Bioscience and Biotechnology Research, University of Maryland, 9600 Gudelsky Drive, Rockville, MD 20850 USA

## Abstract

The 2013–2016 Ebola virus (EBOV) disease epidemic demonstrated the grave consequences of filovirus epidemics in the absence of effective therapeutics. Besides EBOV, two additional ebolaviruses, Sudan (SUDV) and Bundibugyo (BDBV) viruses, as well as multiple variants of Marburg virus (MARV), have also caused high fatality epidemics. Current experimental EBOV monoclonal antibodies (mAbs) are ineffective against SUDV, BDBV, or MARV. Here, we report that a cocktail of two broadly neutralizing ebolavirus mAbs, FVM04 and CA45, protects nonhuman primates (NHPs) against EBOV and SUDV infection when delivered four days post infection. This cocktail when supplemented by the anti-MARV mAb MR191 exhibited 100% efficacy in MARV-infected NHPs. These findings provide a solid foundation for clinical development of broadly protective immunotherapeutics for use in future filovirus epidemics.

## Abstract (web summary)

Current experimental monoclonal antibodies (mAbs) for Ebola virus (EBOV) post-exposure immunotherapy are ineffective against Sudan (SUDV) or Marburg virus (MARV). Here, authors develop cocktails of mAbs that protect nonhuman primates against EBOV, SUDV, and MARV infection when given four days post infection.

## Introduction

The genus *Ebolavirus* consists of five species each represented by a virus type: Ebola (EBOV), Sudan (SUDV), Bundibugyo (BDBV), Reston (RESTV), and Taï Forrest (TAFV) viruses, of which the first three have caused lethality in humans^1^. The 2013–2016 epidemic of EBOV disease (EVD) in western Africa with >28,000 cases and >11,000 deaths is a reminder of the threat these viruses pose to public health. The surface glycoproteins (GP) of ebolaviruses, the primary target of vaccines and immunotherapies, display significant interspecies sequence variability^2^. Additionally, the recent EVD epidemic clearly demonstrated the ability of the virus to mutate during an epidemic^3–5^. Novel therapeutics should target evolutionarily conserved epitopes with a low likelihood of mutations spontaneously or under drug/immune selection pressure. Broadly neutralizing, protective ebolavirus antibodies have, however, seemed elusive until recently^6–14^.

Following endosomal uptake of filoviruses through macropinocytosis, three key steps govern the productive infection of cells: (1) cleavage of GP by cysteine cathepsins to generate cleaved GP (GP_CL_) in which the receptor binding site (RBS) is exposed, (2) GP_CL_ binding to its receptor Nieman–Pick C1 (NPC-1), and (3) fusion with the endosomal membrane and content delivery to the cytosol^15–20^. Previously, we reported on a panel of broadly neutralizing ebolavirus monoclonal antibodies (mAbs). These data indicated that combination of two mAbs, FVM04, and CA45, can block all these three steps^8,11^ (Supplementary Fig. 1A). These chimeric mAbs consist of macaque variable domains fused to human constant regions of IgG1^8,11^. We previously demonstrated efficacy of each individual mAb against EVD in mice^8,11,21^, against SUDV infection in guinea pigs^8,11^, and efficacy of a cocktail of both mAbs against EBOV in guinea pigs and BDBV in ferrets^11^.

In the present study we show the ability of FVM04 and CA45 to bind to a wide range of GP variants that emerged during the 2013–2016 EVD outbreak as well as previously identified mutants of ebolavirus GP that allow escape from several neutralizing antibodies. Based on these properties and the broad reactivity toward all pathogenic ebolaviruses, these mAbs represent excellent candidates for an effective pan-ebolavirus (PE) therapeutic cocktail. We report that this PE cocktail, when delivered post-exposure to nonhuman primates (NHPs) infected with Ebola or Sudan viruses, provides 100% protection. In addition, we demonstrate that a neutralizing mAb against the distant Marburg virus can be added to this cocktail to formulate a pan-filovirus (PF) immunotherapeutic cocktail. The PF antibody cocktail also provides 100% postexposure protection against infection with Ebola, Sudan, and Marburg viruses in guinea pigs and NHPs. These data establish a critical proof of concept for feasibility of PF or PE immunotherapy.

## Results and Discussion

### Binding characteristics of the PE antibodies

The ability of antibodies to bind to GP at acidic pH has been identified as important for neutralization of ebolaviruses^11,22^. We evaluated the binding of FVM04, CA45, and several other previously reported mAbs toward GP of EBOV, SUDV, and BDBV at neutral and acidic pH (pH 4.5). Both mAbs exhibited subnanomolar binding EC_50_ to the GPs at pH 4.5 (Supplementary Fig. 1B). Several GP mutations have been described that lead to loss of binding to the known EBOV neutralizing mAbs: the RBS mutation G118A for FVM04^8^; the base mutation R64A for CA45^11^; Q508A for 2G4 and KZ52; G528E for PE mAbs Adi-15878 and Adi-15742^9^; N550A for CA45^11^, KZ52 and 2G4^23^; D552A for KZ52 and 4G7^23^, and finally H628N for Adi-16061^9^, which binds the membrane proximal stalk region of GP. To assess our proposed cocktail of mAbs, we produced these mutant GP ectodomains and evaluated their binding to FVM04 and CA45, along with a panel of therapeutic candidate mAbs by biolayer interferometry. As shown in Supplementary Fig. 1C, all mutations in the base of GP trimer as well as G118A reduced the affinity of CA45 for GP. However, FVM04 exhibited similar or higher binding affinity to the base mutations as compared to wild-type GP. These data indicated that the vast majority of mutations that result in loss of binding to base and stalk antibodies can be recognized by a cocktail of CA45 and FVM04.

**Fig. 1:**
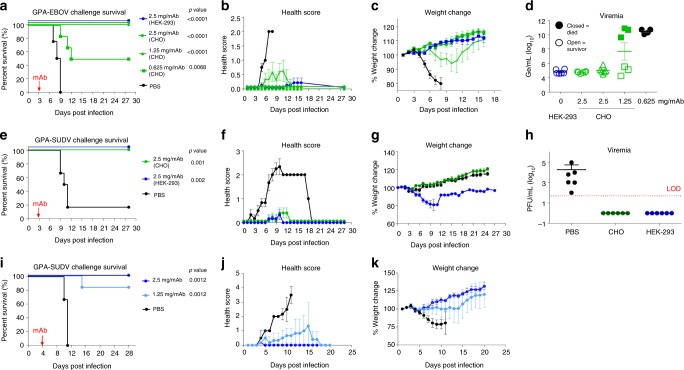
Efficacy of the PE cocktail in guinea pigs against EBOV and SUDV. **a** Survival, **b** health score, and **c** weight change in animals challenged with guinea pig-adapted EBOV (GPA-EBOV) and **d** day 7 viremia data, determined by RT-qPCR. Control group (*n* = 4) was given PBS while treatment groups (*n* = 6) received antibodies on day 3 postinfection. **e** Survival, **f** health score, **g** weight change, and **h** viremia by plaque assay (individual animals and mean ± SD) from animals (*n* = 6) challenged with guinea pig-adapted SUDV (GPA-SUDV) and treated 3 days postinfection or control animals given PBS (*n* = 6), LOD limit of detection. **i** Survival, **j** health score, and **k** weight change in animals (*n* = 6) treated 4 days postinfection with guinea pig-adapted SUDV (GPA-SUDV) or control animals (*n* = 6) given PBS. CHO (green) or 293T (blue) represent the cell line in which antibodies were produced. Error bars represent the mean value of the six animals in each group ± standard deviation (SD). Statistical analysis of survival was performed by log-rank test

FVM04 and CA45 were produced in Chinese Hamster Ovary (CHO) cells and tested for potency in a model of EBOV infection in guinea pigs in which a single dose of the antibody cocktail is administered at 3 days postinfection (dpi) with the guinea pig-adapted (GPA) Ebola virus (GPA-EBOV). CHO-produced material was also compared with antibodies produced in human embryonic kidney cells (HEK-293T). Both CHO and HEK-293T produced material fully protected guinea pigs at 2.5 mg/mAb/animal (Fig. 1a). Doses as low as 1.25 mg/mAb/animal provided full protection (*p* < 0.0001; log-rank test) with little or no sign of disease, while treatment with 0.625 mg/mAb/animal afforded 50% survival (*p* = 0.0068; log-rank test) (Fig. 1a, b). Control animals lost over 10% of their body weight before death while, by contrast, animals treated with 2.5 or 1.25 mg cocktail gained weight; furthermore, only a transient weight loss was observed in the 0.625 mg dose group (Fig. 1c). All surviving animals had<10^6^ genomic equivalents per milliliter (Ge/mL) virus in their blood at 7 dpi (Fig. 1d), while animals that succumbed to death exhibited 4–5 logs higher viremia, suggesting that antibody control of viral replication is key to preventing mortality. Furthermore, the cocktail administered at 2.5 mg/mAb on 3 or 4 dpi (*n* = 6) fully protected (*p* = 0.001 and *p* = 0.0012; log-rank test) against challenge with GPA-SUDV virus compared to controls treated with phosphate-buffered saline (PBS) (Fig. 1e–k).

The sequence identity between ebola- and Marburg virus GPs is <30%^24^. We previously reported several nonneutralizing mAbs reactive to all filoviruses^21,25,26^, however, to-date, cross-neutralizing epitopes between these two viruses that would be accessible on the viral surface have not been identified. Thus, a practical option for a PF immunotherapeutic may be to blend a Marburg virus-specific mAb with a PE cocktail. Mire et al. reported protection against MARV and Ravn virus (RAVV) challenge in NHPs^27^ by a single neutralizing mAb, MR191, which was isolated from a human survivor^28^. We produced MR191 in ExpiCHO cells and generated a new cocktail of FVM04, CA45, and MR191 (PF cocktail). The PF cocktail (2.5 mg CA45 + 2.5 mg FVM04 + 10 mg MR191) was equally protective against MARV challenge with GPA-MARV in guinea pigs treated with 5 or 10 mg of MR191 alone given 3 dpi (Supplementary Fig. 2A, B) and the addition of MR191 did not affect protection against GPA-SUDV challenge when administered 3 dpi (Supplementary Fig. 2C, D), indicating that there is no interference between the two classes of antibodies when combined.

### Efficacy of PE and PF cocktails in NHP EBOV model

**Fig. 2:**
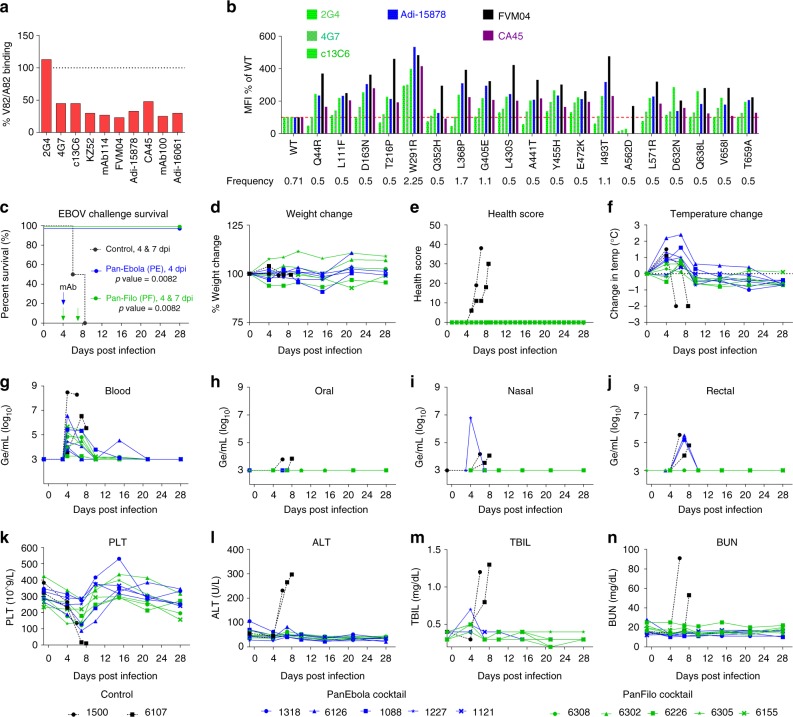
Efficacy of the PE and PF cocktails against EBOV Makona. **a** Binding of the indicated antibodies to V82 GP variant as percentage of binding to A82 Makona GP. **b** Relative binding of the indicated mAbs to Makona quasispecies compared to wt. **c**–**f** Efficacy of PE (*n* = 5) and PF (*n* = 5) cocktails in NHP model of EBOV Makona infection. **g**–**j** Viral loads in blood, as well as oral, nasal, and rectal swabs. **k**–**n** Select hematology and blood chemistry data at various times post challenge. PLT platelets, ALT alanine transaminase, TBIL total bilirubin, BUN blood urea nitrogen. Statistical analysis of survival was performed by log-rank test

While we have previously shown that the PE cocktail neutralizes all pathogenic ebolaviruses^11^, the activity of these mAbs against the Makona variant of EBOV (EBOV/Mak) that caused the 2013–2016 EVD epidemic was not formally tested. Makona GP exhibits a number of amino acid differences from the Mayinga and Kikwit variants, mostly in the mucin-like domain^5^. However, one mutation (A82V), acquired early in the epidemic, and absent in the reference virus H.sapiens-wt/GIN/2014/Makona-Kissidougou-C15 (GenBank# KJ660346.2), is of particular interest as it is located in the RBS and its emergence resulted in enhanced infectivity of human cells by EBOV/Mak^4^. To evaluate the impact of this mutation on binding to our antibodies we expressed the V82 and A82 variants of Makona GP in HEK-293T cells and evaluated binding of a panel of EBOV-specific and PE anti-GP mAbs by flow cytometry. Interestingly, all antibodies tested except for 2G4 exhibited ≥50% reduction in binding to the V82 as compared to A82 Makona GP variants (Fig. 2a) suggesting that this mutant may have evolved early in the epidemic under immunological pressure.

Analyzing 179 genomes isolated from the western Africa EVD epidemic patients between July 2014 and January 2015, Carroll et al.^3^ showed a number of GP sequence variants, that besides the dominant A82V mutation (70% frequency), included several additional substitutions at low frequency of 0.5–2% (Fig. 2b). We expressed these individual mutants on the background of EBOV/Mak-A82V (H.sapiens-wt/SLE/2014/Makona-S57; bearing A82V mutation) in HEK-293T cells and evaluated the binding of a panel of mAbs. Interestingly, most of these mutations increased binding of GP to CA45, FVM04, and the PE mAb Adi-15878 compared to GPwt (Fig. 2b). However, one mutant A562D completely lost binding to CA45 and Adi-15878 and exhibited marginal binding to 2G4, 4G7, and 13C6 (Fig. 2b). In contrast, FVM04 retained binding to A562D at a level higher than GPwt (Fig. 2b). These data indicate that a cocktail of FVM04 and CA45 (PE cocktail) retains binding to all observed variants of Makona GP.

The efficacy of PE and PF cocktails against EBOV/Mak was evaluated in rhesus macaques. Twelve macaques, randomly assigned to group 1 (*n* = 2) and groups 2–3 (*n* = 5 each), were infected with 1000 × TCID_50_ (actual dose delivered: 734 TCID50) of EBOV/Mak-C07 (H.sapiens-wt/GIN/2014/Makona-Gueckedou-C07) (an A82 variant) via intramuscular (IM) route. At 4 dpi, group 2 received PE cocktail (20 mg/kg/mAb) and group 3 received PF cocktail (20 mg/kg FVM04 and CA45, and 50 mg/kg MR191) via intravenous (IV) route. Group 3 also received a second dose of PF cocktail (10 mg/kg PE mAbs, and 50 mg/kg MR191) at 7 dpi. Group 1 animals were treated with vehicle at 4 and 7 dpi as controls. Animals were monitored for signs of disease for 28 days.

The control animals showed rapid deterioration of their health score between days 4–8 while no clinical sign of disease was observed in treated animals. Observation variables included responsiveness and recumbence, food consumption, condition of stool, presence of urine output, respiratory difficulty, as well as the presence of rash, bleeding, cyanosis, hemorrhage, or exudates (Fig. 2e). Modest fever but little weight change was observed in most animals (Fig. 2d, f). Blood was collected on days 0, 4, 7, 10, 15, 21, and 28 for analysis.

All animals were viremic at 4 dpi, as determined by RT-qPCR (Fig. 2g), and the two controls succumbed to infection on days 6 and 8, while all animals in groups 2 and 3 survived the challenge (*p* = 0.0082; log-rank test) (Fig. 2c). Day 4 viremia varied widely between the animals. Of the two controls NHP-1500 showed peak viremia of 3 × 10^8^ Ge/mL on day 4, while NHP-6107 was mildly viremic on day 4 (3.7 × 10^3^ Ge/mL) but peaked on day 6 at 3.7 × 10^6^ Ge/mL before dying on day 8. Pretreatment viral titers in groups 2 and 3 ranged from 1.8 × 10^3^ Ge/mL (NHP-6226, group 3) to a maximum of 3.7 × 10^6^ Ge/mL (NHP-6126 Group 2). All ten antibody-treated animals showed a rapid decline and complete clearance of the virus in peripheral blood. One animal (NHP-6126) exhibited a brief spike of 3.5 × 10^4^ Ge/mL on day 15 but went back to undetectable by day 21. Nasal and rectal swabs were positive in most animals, while low oral titers were seen only in control animals on the day of death (Fig. 2g–j).

Control animals exhibited drastic thrombocytopenia before death, while the treated animals showed mostly a transient reduction in platelets, which rebounded after treatment (Fig. 2k). Additional hematological changes included transient reduction in monocytes and lymphocytes as well as an increase in neutrophils on day 4 (Supplementary Fig. 3). Both control NHPs exhibited high levels of alanine aminotransferase (ALT) and total bilirubin (Fig. 2l, m), as well as alkaline phosphatase (ALP) (Supplementary Fig. 3), indicating liver damage, while liver function remained normal in antibody-treated animals. Kidney damage was evident by sharp increase in circulating blood urea nitrogen (BUN) (Fig. 2n) and to a lesser extent creatinine (CRE) (Supplementary Fig. 3) in controls but not treated animals. Additional hematology and blood chemistry data are provided in Supplementary Fig. 3.

To examine if the infection resulted in an immune response to EBOV we measured serum IgM and IgG levels throughout the study. The control animals did not show any IgM responses, as they died on days 6 and 8, while all antibody-treated animals showed a robust IgM response of 10–100-fold over baseline peaking at 10 dpi. Anti-GP IgG level rose rapidly between 6 and 10 dpi, primarily due to the administered IgG that is indistinguishable from macaque IgG in this assay (Supplementary Fig. 3H–K). IgG titers continued to rise beyond day 10 in all treated animals as IgM subsided suggesting high level of endogenous IgG production from macaque cells. These data clearly indicate that treatment allows for the endogenous immune response to initiate and possibly contribute to protection.

### Efficacy of PE and PF cocktails in NHP SUDV model

**Fig. 3:**
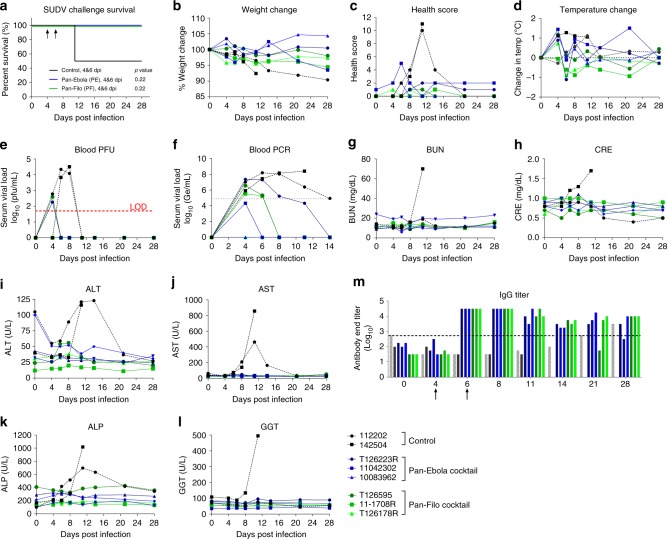
Efficacy of PE and PF cocktails against SUDV infection. Rhesus macaques were infected with SUDV and treated with PE (*n* = 3) or PF (*n* = 3) cocktails at 4 and 6 dpi. Survival (**a**), daily health evaluations (**b**–**d**), viremia by plaque assay (limit of detect of 50 pfu/mL) (**e**) and RT-qPCR (**f**), blood chemistry (**g**–**l**), and serology data (**m**) are shown. BUN blood urea nitrogen, CRE creatine, ALT alanine aminotransferase, AST aspartate aminotransferase, ALP alkaline phosphatase, GGT gamma-glutamyl transferase. Statistical analysis of survival was performed by log-rank test

Eight rhesus macaques were randomly assigned to a control group (*n* = 2) and two treatment groups (*n* = 3). On day 0, all NHPs were challenged with a target dose of 1000 plaque-forming units (PFU) of SUDV/Bon via the IM route (actual dose delivered: 2625 PFU). Group 1 received vehicle control, and group 2 received PE cocktail at 4 dpi (20 mg/kg/mAb) and 6 dpi (8 mg/kg FVM04 and 5 mg/kg CA45). Group 3 received PF cocktail at 4 and 6 dpi by IV route with the same dose of PE antibodies as group 2 plus 50 mg/kg of MR191. One vehicle treated animal succumbed to disease on day 11 following exposure (Fig. 3a). All other animals survived the challenge. No change of weight over 10% was observed in any of the NHPs and no animal showed more than 1.5 °C fluctuation in body temperature (Fig. 3b, d). The clinical scoring was determined by cage side observation and included ruffled fur, slowing of activity, labored breathing, hunched posture, and bleeding. Four mAb treated animals remained near baseline throughout the study, except for a transient increase in one animal at 6 dpi. Both controls exhibited clinical signs of disease, as reflected by their high-clinical scores, which resolved in the surviving control by 21 dpi (Fig. 3c). Blood draws were performed on days 0, 4, 6, 8, 11, 14, 21, and 28 for analysis. Control animals developed viremia by day 4 that resolved by day 11 as determined using plaque assay, though it remained positive by RT-qPCR through day 14 for the surviving control NHP (Fig. 3e, f). One animal in each treatment arm was viremic according to plaque assay at 4 dpi; viremia resolved by 6 dpi. All but two animals were viremic by RT-qPCR at initiation of treatment, but cleared the virus by days 6, 8, or 14. Gross pathology analysis of NHP-142504, which died on day 11, showed lesions typical of SUDV infection while gross lesions that may be attributed to SUDV infection were only observed in a single treated animal (T126223R).

Blood chemistry analysis indicated kidney damage in the control NHP that succumbed, while liver damage was evident by day 10 in both control animals. All antibody-treated animals showed normal levels of the markers tested (Fig. 3g–l). Complete blood counts (CBC) were measured from whole blood at multiple time points. Lymphopenia was observed in a single control on day 8 after exposure. Both control animals experienced thrombocytopenia at multiple time points, while two experimental animals exhibited thrombocytopenia on day 4 before returning to normal.

All animals remained near or below the limit of detection for SUDV-specific serum IgG through 4 dpi (Fig. 3m). Following the Day 4 treatment, there was an expected spike, due to antibody administration, which remained through day 8 following exposure. SUDV-specific IgG titers decreased somewhat for treated animals through the completion of the study, while rising for the surviving control animal, consistent with a longer and higher level of viremia in this animal.

### Efficacy of PF cocktail against MARV infection in NHPs

**Fig. 4:**
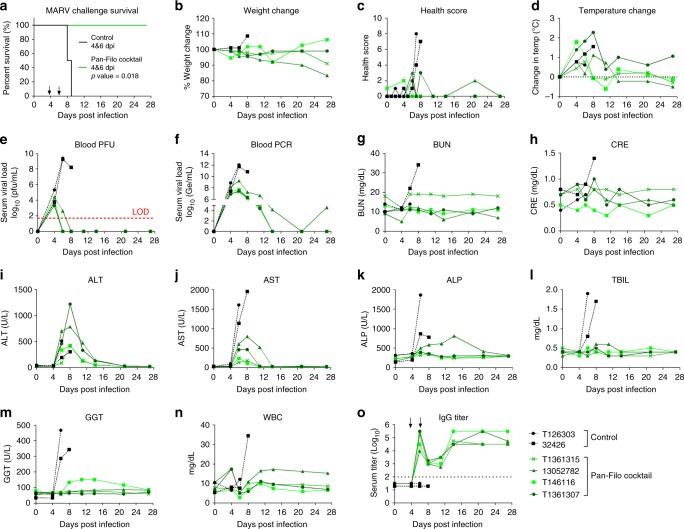
Efficacy of PF cocktail against MARV infection. Rhesus macaques were infected with MARV (Ci67) and treated with the PF cocktail (*n* = 4) at 4 and 6 dpi. Survival (**a**) daily health evaluations (**b**–**d**), viremia by plaque assay (limit of detect of 50 pfu/mL) (**e**) and RT-qPCR (**f**), blood chemistry (**g**–**m**), white blood cells (**n**), and serology data (**o**) are shown. CRE creatine, AST aspartate transaminase, ALP alkaline phosphatase, GGT gamma-glutamyl transferase, WBC white blood cells. Statistical analysis of survival was performed by log-rank test

The above results demonstrated that presence of an anti-MARV antibody does not interfere with efficacy against EBOV and SUDV. We next evaluated the efficacy of the PF cocktail in a rhesus macaque model of MARV infection. Six animals were infected with a target dose of 1000 PFU of MARV (variant Ci67). Animals received vehicle control (*n* = 2) or PF cocktail (*n* = 4), on 4 dpi (20 mg/kg of CA45 and FVM04 + 50 mg/kg MR191) and 6 dpi (10 mg/kg of CA45 and FVM04 + 50 mg/kg MR191) and were monitored for 28 days postexposure. Both control animals displayed clinical signs of disease and succumbed to infection on days 8 and 9, while all antibody-treated animals survived (*p* = 0.018; log-rank test) (Fig. 4a–d). Histopathologic lesions consistent with Marburg virus disease were observed in the liver, spleen, lymph nodes, and reproductive organs of both control animals (Supplementary Fig. 4). Minimal and/or nonspecific histologic lesions were observed in all surviving animals. MARV antigen was detected in the liver and spleen of both control animals and was not detected in the liver or spleen of any survivors. Three NHPs developed fever, two of the mAb treated animals and a single vehicle-treated control (Fig. 4d). Blood draws were performed on days 0, 4, 6, 8, 11, 14, 21, and 27 for analysis. All animals were viremic as determined by plaque assay at the initiation of treatment on day 4 (Fig. 4e, f). While the treated animals cleared the virus by day 8 (based on plaque assay) or 21 (based on RT-qPCR), the viremia continued to rise in control animals to reach nearly 1 × 10^10^ PFU/mL before death. RT-qPCR analysis showed that one of the PF cocktail-treated animals (13052782) that first cleared the virus by day 21 was positive by RT-qPCR (~1 × 10^5^ Ge/mL) at 27 dpi prior to study termination (Fig. 4f). This animal also showed a health score of 2 on day 21 and continued to lose weight through day 27 (Fig. 4c). However, no infectious virus could be detected in the blood of this animal beyond 8 dpi.

Increased BUN and CRE in blood were observed in one of the control animals (NHP 32426) (Fig. 4g, h), while enzyme levels indicated severe liver damage in both controls (Fig. 4i–m). Treated animals exhibited transient increases in ALT, ALP, and aspartate transaminase, but the values returned to normal as the animals recovered from the disease (Fig. 4i–k). Additional hematology and blood chemistry data are provided in Supplementary Fig. 5. Similar to the ebolavirus challenge studies the animals mounted an immune response to the infection as evidenced by a rise in endogenous IgG titers by day 14 (Fig. 4o). These data are the first to demonstrate protective efficacy of MR191 as part of a PF cocktail against MARV in the NHP model of disease.

In summary, the PE therapeutic antibody cocktail presented here provides broad neutralization of all three pathogenic ebolaviruses which have caused fatal disease in humans. By blocking nonoverlapping epitopes in the apex and the base of the GP trimer, the cocktail blocks three critical steps of viral entry. The combination allows for reactivity to those potential mutants that may have lost binding to one class of antibodies, providing increased confidence that viral escapes can be avoided. Furthermore, for the first time, our studies demonstrate the feasibility of a three antibody cocktail for protection against both ebolavirus and Marburg virus infections. Since these antibodies are human-macaque chimeric mAbs, all effector functions are therefore mediated by the fully human Fc domains of these antibodies. While human and macaque antibody variable domains genes are 91 ± 2.2% identical in amino acid sequences^29^, potential immunogenicity of such antibodies cannot be entirely ruled out. However, given the acute nature of filovirus infection these anti-filovirus immunotherapeutics would be used for a short period of time minimizing the risk of complications resulting from treatment with these mAbs. Future humanization of the chimeric mAbs can further reduce this minimal risk. These findings provide a solid foundation for development of immunotherapeutics for protection of humans against multiple filoviruses during an epidemic.

## Methods

### Regulatory statement

Animal research performed at US Army Medical Research Institute of Infectious Diseases (USAMRIID) was conducted under a protocol approved by the USAMRIID Institutional Animal Care and Use Committee (IACUC) in compliance with the Animal Welfare Act and other federal statutes and regulations relating to animals and experiments involving animals. The USAMRIID facility is fully accredited by the Association for the Assessment and Accreditation of Laboratory Animal Care International and adheres to the principles stated in the Guide for the Care and Use of Laboratory Animals, National Research Council, 2011. Challenge studies are conducted under maximum containment in an animal biosafety level 4 facility.

Animal studies performed at the National Microbiology Laboratory (NML) in Winnipeg, Manitoba, Canada have been approved by the Animal Care Committee at the Canadian Science Center for Human and Animal Health in accordance with the guidelines outlined by the Canadian Council on Animal Care.

### Ebola adapted guinea pig challenge

Female Hartley guinea pigs 4–6-week old, were randomly assigned to four groups (*n* = 6) and one control PBS group (*n* = 4) were challenged with 1000× LD50 via intraperitoneal (IP) route with GPA-EBOV. Animals were observed daily and given a score from 0 to 4, with a score of 4 requiring euthanasia. CHO derived FVM04 + CA45 cocktail was administered at 3 dpi with material produced in HEK-293T cells used as reference standard. The cocktail was given at 2.5 mg/Ab, 1.25 mg/Ab, and 0.625 mg/Ab.

### Sudan adapted guinea pig challenge

Female Hartley guinea pigs at 4–6-weeks old were obtained from Charles River (Wilmington, MA), divided into three groups (*n* = 6) for each experiment and challenged IP with a target dose of 1000 PFU of GPA-SUDV. MAbs were administered IP 3 dpi at 2.5 mg/Ab per guinea pig or 4 dpi at 2.5 or 1.25 mg/Ab. Control animals received an equivalent volume of vehicle PBS. Individual weights were recorded daily through the end of each study. Animals were observed daily for clinical signs of disease; observations were increased to twice daily upon onset of clinical signs of disease. Moribund and surviving animals were humanely euthanized in accordance with IACUC-approved criteria.

### Ebola NHP challenge

EBOV NHP challenge experiments took place at the Public Health Agency of Canada (PHAC) at the NML in Winnipeg, MB, Canada. This study was not blinded, and animals were assigned into groups based on gender and weight. Upon arrival at the NML, NHPs were transported to an animal biosafety level (ABSL)-2 containment animal room and housed according to approved animal protocol. The NHPs were housed in a temperature-monitored and humidity-monitored room. Water was provided ad libitum via an automated watering system. The temperature was 69–70 °F and the humidity varied from 24 to 59%. Automated light controls were used for light/dark cycles of approximately 12 h. Animals were provided commercially available animal feed (monkey chow) at least daily. Fruits, vegetables, and treats were also provided at least three times per week. Procedures involving manipulation, including phlebotomy or physicals were performed while animal was under anesthesia. Acclimation in the ABSL-2 environment lasted for 7 days before infection.

Twelve NHP rhesus macaques (*Macaca mulatta*) were randomly assigned into groups (A–C) based on gender and weight. Group A: A1-1500 (F), A2-6107(M); Group B: B1-1318 (F), B2-6126 (F), B3-1088 (M), B4-1227 (M), B5-1121 (M); Group C: C1-6308 (F), C2-6302 (F), C3-6226 (F), C4-6305 (M), C5-6155 (M). All NHPs were challenged with a dose of 1000× TCID_50_ via IM route. The challenge virus used in NHPs was EPOV H.sapiens-wt/GIN/2014/Makona-Gueckedou-C07 (EBOV-C07) (order Mononegavirales, family Filoviridae, species *Zaire ebolavirus*; GenBank accession no. KJ660347.2). Four days postinfection, Group B animals were given a 20 mg/kg (FVM04 + CA45) IV bolus of antibody cocktail (*n* = 5 per group); Group C animals were given a 20 mg/kg (FVM04 + CA45) plus 50 mg/kg MR191 IV bolus of antibody cocktail (*n* = 5 per group); 7 days postinfection, group C animals were given a 10 mg/kg (FVM04 + CA45) plus 50 mg/kg MR191 IV bolus of antibody cocktail (*n* = 5 per group). Group A animals (*n* = 2) were given similar volume of vehicle control (20 mM citrate, 10 mM glycine, 8% sucrose, 0.01% polysorbate-80 at pH 5.5) at 4- and 7-days postinfection delivered via slow syringe push lasting 3–5 min intravenously via the saphenous veins.

After exposure to EBOV, NHPs were observed at least twice daily until onset of clinical signs at which time the NHPs were observed at least three times daily. All daily observations were documented on the NHP Humane Endpoint Clinical Scoring Chart. Observation variables included responsiveness and recumbence, food consumption, condition of stool, presence of urine output, respiratory difficulty, as well as the presence of rash, bleeding, cyanosis, hemorrhage, or exudates. On dates of blood collection (days 0, 4, 7, 10, 15, 21, and 28), the NHPs were anesthetized and a physical examination was performed by qualified study personnel. Weight and body temperature measurements were also collected at this time. Specimens collected throughout the study were inventoried and stored under appropriate (both containment and sample specific) conditions at −70 ± 10 °C.

### Sudan and Marburg NHP challenge

Healthy, filovirus-naive rhesus macaques (*Macaca mulatta*) were obtained from the available colony at USAMRIID. Pre-exposure physicals, complete blood counts and chemistries were performed under anesthesia on day 7, immediately prior to BSL-4 transfer. For Sudan virus challenge experiments, 5 females (112202 (control), 11042302, 10083962, 11-1708 R, T126178R) and 3 males (142504 (control), T126223R, T126595) were randomly assigned into 3 treatment groups: vehicle (*n* = 2), PE cocktail (*n* = 3), or PF cocktail (*n* = 3). Challenge doses were prepared in 0.9% saline. NHPs were injected intramuscularly in the left quadriceps with a target dose of 1000 PFU of the Boniface isolate (SUDV/Bon) in a volume of 0.5 mL. Actual dose was determined by subsequent plaque assay to be 2625 PFU/NHP. Four- and six-days following challenge, animals were treated IV with vehicle (20 mM citrate, 10 mM glycine, 8% sucrose, 0.1% polysorbate-80, at pH 5.5) (group 1), PE cocktail (group 2) or PF cocktail (group 3). On day 4, PE was delivered at a dose of 20 mg/kg/Ab; PF consisted of PE plus MR191 at 40 mg/kg. On day 6, PE was delivered at a target dose of 10 mg/kg/Ab (actual dose determined to be 8 mg/kg/Ab FVM04 and 5 mg/kg/Ab CA45); PF consisted of the same dose of PE plus MR191 at 40 mg/kg. All treatment volumes were normalized against animal weight.

For Marburg virus challenge experiments, six rhesus macaques, three females (T146116, 13052782, and 32426) and three males (T1261303, T1361307, and T1361315), were randomized into two treatment groups: vehicle control (*n* = 2) and PF (*n* = 4). Animals were challenged IM with a target dose of 1000 PFU of the Ci67 isolate of MARV. Actual dose was determined by subsequent plaque assay to be 969 PFU/NHP. Four days after infection, four animals were treated with the PF cocktail at 20 mg/kg of CA45 and FVM04 + 50 mg/kg MR191, while two control animals were given vehicle (20 mM citrate, 10 mM glycine, 8% sucrose, 0.1% polysorbate-80, at pH 5.5). All treatments were delivered IV, alternating sides per treatment. Animals were treated again on day six postinfection with the cocktail containing an equivalent dose of MR191, while halving the dose of the PE component. Control animals received a second vehicle treatment. All treatment volumes were normalized to animal weight.

### Morbidity and mortality

For Ebola NHP challenge studies all observations, including when animals appeared normal, were recorded on approved forms. Animals judged moribund by a trained animal technician, Staff Veterinarian, or Study Director, had blood collected prior to euthanasia with an overdose of a euthanasia agent containing pentobarbital. Moribund animals were euthanized when they met secondary euthanasia criteria following a primary euthanasia criterion of score of 25. Animals were euthanized by qualified staff by administration of euthanasia solution (Fatal Plus^®^). Death was verified by the absence of a heartbeat at no fewer than 5 min. Body temperatures were obtained via rectal monitoring on dates of blood collection and at the time of euthanasia. Animals were weighed on scheduled dates of samples collection (days 0, 4, 7, 10, 15, 21, and 28) for which the animals were anesthetized for routine phlebotomy, physical examinations, and euthanasia.

For Sudan and Marburg challenge studies, NHPs were observed cage side daily for clinical signs of disease. When animals displayed clinical signs, observations were increased to a minimum of twice daily, with no less than 4 h between observations. Physical observations and blood draws were performed at regular intervals following and including the day of challenge. On these days, anesthetized animals were assessed more closely for signs of disease to include weight, temperature, lymph node swelling, petechial rash, edema, and dehydration. Whole blood, plasma, and serum were collected following blood draws on days 0, 4, 6, 8, 11, 14, 21, and 27 (MARV) or 28 (SUDV) for further evaluation.

### Hematology and blood chemistry

For Ebola NHP challenge studies samples of whole blood were collected in liquid ethylenediaminetetraacetic acid (K_3_EDTA) and serum tubes. CBCs were performed with the VetScan HM5 (Abaxis Veterinary Diagnostics). Blood biochemistry analyses were performed with the VetScan VS2 (Abaxis Veterinary Diagnostics). For Sudan and Marburg challenge studies whole blood specimens were collected in EDTA tubes and analyzed using a Beckman Coulter AcT 10 hematology analyzer for CBCs. Serum was separated from whole blood specimens using serum separator tubes and chemistries were analyzed using a Piccolo Xpress Chemistry Analyzer (Abbott).

### Viremia determination by TCID_50_ and RT-qPCR

Titration of EBOV was performed as according to the following procedue^30^. Supernatants or virus from whole blood were serially diluted tenfold in Dulbecco’s modified media (DMEM). Vero E6 (ATCC, Cat# CRL-1586) cells (at 80% confluence in a 96-well plate (Corning)) were then inoculated with 100 µL of each dilution, in triplicate, and cells were incubated at 37 °C for 1 h. Following incubation, the supernatant was removed and replaced with 100 µL of fresh DMEM with 2% fetal bovine serum (FBS). Cells were incubated for 14 days, and then scored for the presence of cytopathic effects. Titers were calculated by the Reed and Muench method. Results are expressed as TCID_50_/mL.

RNA levels were measured by RT-qPCR according to the following protocol^30^.Total RNA was extracted from whole blood, nasal swabs, oral swabs and rectal swabs with the QIAmp Viral RNA Mini Kit (Qiagen). EBOV was detected with the LightCycler 480 RNA Master Hydrolysis Probes (Roche) kit, with the RNA polymerase (nucleotides 16,472–16,538, AF086833) as the target gene. The reaction conditions were as follows: 63 °C for 3 min, 95 °C for 30 s, and cycling of 95 °C for 15 s, 60 °C for 30 s for 45 cycles on the Applied Biosystems Quant Studio 3. The lower detection limit for this assay is 86 genome equivalents per ml. The sequences of primers and probe used were as follows: EBOVLF2(CAGCCAGCAATTTCTTCCAT), EBOVLR2 (TTTCGGTTGCTGTTTCTGTG), and EBOVLP2FAM (FAM-ATCATTGGCGTACTGGAGGAGCAG-BHQ1).

For SUDV and MARV NHP challenge, plaque assays were performed based on the following procedure^31^. NHP serum was serially diluted in growth media, added to VeroE6 cells and incubated for 1 h at 37 °C in a humidified 5% CO_2_ incubator, then overlaid with a preparation of 2× Eagle’s Basal medium (Gibco), 10% FBS (HyClone), and 1% SeaKem ME agarose (Lonza). Infected cells were incubated for 7 days, then stained with neutral red vital dye (Gibco). Plaques were counted, and titers were calculated. For RT-qPCR, performed by the USAMRIID PCR Core Laboratory, serum or plasma was mixed 1:3 with Trizol LS (Thermo Fisher) and sample was extracted using the QIAamp Viral RNA Mini Kit in accordance with manufactures guidelines and USAMRIID Standard Operating Procedures. Synthetic RNA representative of the target region of the SUDV or MARV was diluted in RNase free water was used to generate an eight-point standard curve for the quantitative determination of virus concentration using the Applied Biosystems 7500 Fast Dx System. The Qiagen QuantiFast Internal Control RT-qPCR assay was used to monitor PCR inhibition and extraction integrity. The endpoint of the assay was reported as genomic equivalents per PCR reaction (Ge/rxn) of SUDV or MARV virus in test samples. The concentration of unknown samples was determined based on the standard curve run on the same plate. Final data is reported at Ge/mL.

### Serum IgG and IgM ELISA

For EBOV, SUDV, and MARV challenge studies, IgG and IgM levels were measured by enzyme-linked immunosorbent assay (ELISA). For EBOV, plates were coated overnight with EBOV GPΔTM (37.5 ng/well) at 4 °C. Goat anti-human IgG/IgM-horseradish peroxidase (HRP) and 3,3′,5,5′- tetramethylbenzidine (TMB) substrate were used to detect the captured IgG and IgM from the samples. For SUDV and MARV challenge studies, blood serum samples and naive NHP serum were serially diluted 1:2 and added to plates coated with recombinant MARV or SUDV glycoprotein antigen. Following room temperature incubation, plates were washed and incubated with 1:2000 dilution of goat anti-human-HRP (KPL). Plates were washed and incubated with 2,2′-azinobis [3-ethylbenzothiazoline-6-sulfonic acid]-diammonium salt substrate. Absorbance values were measured at 405 nm using a SpectraMax M5 plate reader (Molecular Devices, Sunnyvale, CA) with SoftMax Pro acquisition software. Cut-off values were determined using negative control serum from a filovirus naive NHP and the following formula: average naive NHP absorbance + 3× the standard deviation. The end-point titer is reported as the last dilution to exceed the cut-off value for a given dilution.

### Pathology

Necropsies of each animal were performed by board-certified veterinary pathologists from the Division of Pathology and gross findings were recorded. The following tissues were collected at necropsy for histology processing: inguinal lymph node, axillary lymph node, spleen, liver, and kidneys. On select animals, tissues with gross lesions were sampled. Tissues were fixed in 10% neutral buffered formalin for a minimum of 30 days prior to removal from containment. After fixation, samples were trimmed, processed, and embedded in paraffin. Five µm sections of the paraffin-embedded tissues were cut for histology. Histology slides were processed to remove residual paraffin and stained with hematoxylin and eosin (H&E), cover-slipped and labeled.

### Antibody production

Plasmids encoding the IgH and IgL of FVM04, CA45, or MR191 were transiently transfected with Expifectamine using the ExpiCHO system (Thermo Fisher, Cat# A29133) according to manufactures’ recommendations. Cells were cultured for 14 days before harvesting the supernatant containing IgG. Supernatant was clarified by centrifugation at 3000 rpm for 10 min at 4 °C and sterile filtered using a 0.2 µm pore sized filer device. Antibodies were purified according to the following procedure^11,21^. Antibodies were captured by HiTrap protein A (catalog no. 17-0403-01; GE Healthcare), washed, and eluted with 0.1 M glycine buffer (pH 2.4). Fractions containing the Ab peak were collected, neutralized with Ab buffer (20 mM l-histidine [pH 6.0], 150 mM NaCl, and 4% sucrose) and dialyzed against the same buffer overnight at 4 °C. Fractions containing IgG were verified by sodium dodecyl sulfate polyacrylamide gel electrophoresis (SDS-PAGE) and western blot. Fractions were pooled and dialyzed against 50 mM Tris pH 7.5, 50 mM arginine–glutamic acid, 150 mM sodium chloride, 5% glycerol prior to storage at −80 °C. IgG concentration was quantified using Peirce BGG protein assay kit (Thermo Scientific). IgH and IgL Genbank accession numbers are KY859862 and KY859863 for CA45; EMD6236 for MR191, and FVM04 is published in patent WO2016069627.

### Acidic pH ELISA

ELISAs were performed as according to the following procedure. EBOV GPΔTM, SUDV GPΔTM or BDBV GPΔTM were diluted in 1× Dulbecco’s PBS and coated into 96-well Maxisorb plates overnight at 4 °C. The next day, plates were washed and blocked with StartingBlock (ThermoFisher) for 1 h at room temperature. After blocking, the plates were washed before adding antibodies diluted in either PBS pH 7.4 or sodium acetate pH 4.5. Antibodies were incubated with glycoprotein for 1 h at room temperature before washing and adding 1:4000 Goat-anti-Human-HRP (SeraCare) for 1 h at room temperature. After 1 h, the plates were washed and 100 µL of TMB was added to each well for 30 min before reading on a VersaMax plate reader at an OD of 650 nm. Data were exported to GraphPad prism and fit to a 4 PL curve to establish an EC50.

### EBOV GPΔTM mutants

EBOV GPΔTM Mayinga; residues 18–650 was expressed within p-ExpreS2 vector containing an N-terminal 17 residue BIP secretion signal. The vector was transfected into *Drosophila melanogaster* S2 cells obtained from ExpreS2ion Biotechnologies (Denmark) using Cellfectin II (Invitrogen) as per manufacturers’ protocol. Stable selection of cells was facilitated with 1000–2000 µg/mL of Zeocin (Invitrogen). Cells were harvested by centrifugation every 3–4 days at 4400×*g* for 20 min and the supernatant filtered. EBOV GPΔTM was engineered with a C terminal histidine tag (×10His) for HP His Trap nickel column (GE Healthcare) purification. The column was washed with Buffer A (20 mM sodium phosphate pH 7.4, 0.5 M sodium chloride, 50 mM arginine–glutamic acid, 15 mM imidazole), followed by a linear gradient over 45 column volumes from Buffer A to Buffer A containing 0.3 M imidazole. Fractions containing EBOV GPΔTM verified by SDS-PAGE and western blot, were dialyzed against 50 mM Tris pH 7.5, 50 mM arginine–glutamic acid, 150 mM sodium chloride, 5% glycerol and concentrated to ~1 mg/mL prior to storage at −80 °C. Size-exclusion HPLC using a Superose 6, 10/300 column confirmed the trimeric form of GP (~450 kDa). Seven single-point mutations which abrogate binding to a specific known antibody or to a class of antibodies (stalk, base, IFL, etc.) were selected based on published data as well as available alanine scanning library (provided by Ben Doranz). Mutants R64A (CA45; IFL), G118A (FVM04, mAb114; RBS), Q508A (KZ52, 2G4, 4G7; base binders), G528E (Adi-15742, Adi-15878; tip of IFL), N550A (CA45, KZ52; IFL & Base), D552A (KZ52; base), and H628N (Adi-16061; stalk) were made within the same EBOV GPΔTM construct and purified similarly.

### Biolayer interferometry

All kinetics data were obtained using an Octet Red 96 machine using 96-well plates, shaking at 1000 rpm at 25 °C. Experiments were performed using 1× kinetics buffer (1× PBS, pH 7.4, 0.01% bovine serum albumin, and 0.002% Tween 20) (ForteBio) as the diluent. Prehydrated Protein G sensors (Fortebio) were dipped into 1× kinetics buffer (ForteBio) for 30 s to establish a stable baseline before loading antibodies (1 µg/mL) onto the sensors 120 s. After ligand loading, another baseline in 1× kinetics buffer was established for 30 s before coated sensors were dipped into wells containing EBOV GPΔTM wild-type or mutant proteins. The concentration of analyte protein ranged from 150 to 2.3 µg/mL. The 300 s association step was followed by a 300 s dissociation step. To control for nonspecific binding of glycoprotein to sensors in the absence of antibody, reference sensors were used in parallel for each concentration of glycoprotein and subtracted from total response. Data analyses were performed with ForteBio Data Analysis 9 software. To obtain *k*_on_, *k*_off_, and *K*_D_ values, specific binding curves were fit globally to a 1:1 Langmuir binding model.

### Flow cytometry for binding of mAbs to Makona GP variants

HEK-293T cells (ATCC^®^ CRL-3216) were transfected with Makona GP variants using FuGENE HD following the manufacturer’s instructions. Twenty-four hours later, cells were washed with PBS and detached by using 5 mM EDTA/PBS. The reaction was stopped by adding complete DMEM and gentle pipetting for single cell suspension. Cells were centrifuged at 1200 rpm for 3 min and resuspended in cold PBS buffer with 2% FBS (FACS buffer) and split equally into 96-well V-bottom plates for EBOV antibody staining. Staining was performed in a 100 μL cell suspension with 10 μg/mL primary EBOV antibody at 4 °C. Thirty minutes later cells were washed with 200 μL FACS buffer for three times to remove the nonbinding antibody. Secondary staining was then performed in a 100 μL suspension with 10 μg/mL goat anti-human IgG Phycoerythrin conjugate (SouthernBiotech, cat# 2043-09) at 4 °C in the dark. Thirty minutes later cells were washed three times with 200 μL FACS buffer and then fixed with fresh 2% paraformaldehyde for flow cytometry analysis. A total of 10,000 single cells of each staining was detected for binding of antibodies to Makona GP variants measured by the mean fluorescence intensity of PE.

### Statistical analysis

Kaplan–Meier was used for analysis of differences in survival. *P* value was generated by the Cox–Mantel log-rank test. All analyses were carried out in GraphPad Prism.

### Reporting summary

Further information on experimental design is available in the Nature Research Reporting Summary linked to this article.

## Supplementary information

Supplementary Information

Source Data

Reporting Summary

## Data Availability

The authors declare that the data supporting the findings of this study are available within the article and its Supplementary Information files, or are available from the corresponding authors upon request. The source data underlying Figs. 1a–k, 2a–n, 3a–m, 4a–o, Supplementary Figures 1C–E, 2A–D, 3A–L, and 5A–T are provided as a Source Data file.
