# Supplementary material for: Post-exposure immunotherapy for two ebolaviruses and Marburg virus in nonhuman primates
Source: Nat Commun. 2019 Jan 10;10:105. doi: 10.1038/s41467-018-08040-w (PMC6328579; doi:10.1038/s41467-018-08040-w)
Supplement: Supplementary file 1 — Supplementary Information [file 41467_2018_8040_MOESM1_ESM.pdf]

Supplementary Information

Post-exposure Immunotherapy of Ebola, Sudan, and Marburg virus infection in nonhuman primates

Jennifer M. Brannan[1†], Shihua He[2†], Katie A. Howell[3], Laura I. Prugar[1], Wenjun Zhu[2], Hong Vu[3], Sergey Shulenin[3], Shweta Kailasan[3], Henna Raina[3], Gary Wong [2,4], Md Niaz Rahim [2,4], Logan Banadyga[2], Kevin Tierney [2], Xuelian Zhao[6], Yuxing Li[5,6], Frederick W. Holtsberg[3], John M. Dye[1*], Xiangguo Qiu[2,4*], M. Javad Aman[3*]

Affiliations:

[1]US Army Medical Research Institute of Infectious Diseases, Frederick, MD, USA.

[2]Special Pathogens Program, National Microbiology Laboratory, Public Health Agency of Canada, Winnipeg, MB R3E 3R2 Canada.

[4]Deparment of Medical Microbiology, University of Manitoba, MB R3E 0J9, Canada.

[3]Integrated BioTherapeutics, Inc., Rockville, MD 20850, USA;

[5]Institute for Bioscience and Biotechnology Research, University of Maryland, Rockville, MD 20850, USA.

[6]Department of Microbiology and Immunology, University of Maryland School of Medicine, Baltimore, MD 21201, USA

*Correspondence to:

M. Javad Aman: javad@integratedbiotherapeutics.com

Xiangguo Qiu: xiangguo.qiu@canada.ca

John M. Dye: john.m.dye1.civ@mail.mil

† These authors contributed equally to this work

Integrated BioTherapeutics, Inc.

4 Research Ct., Suite 300

Rockville, MD 20850

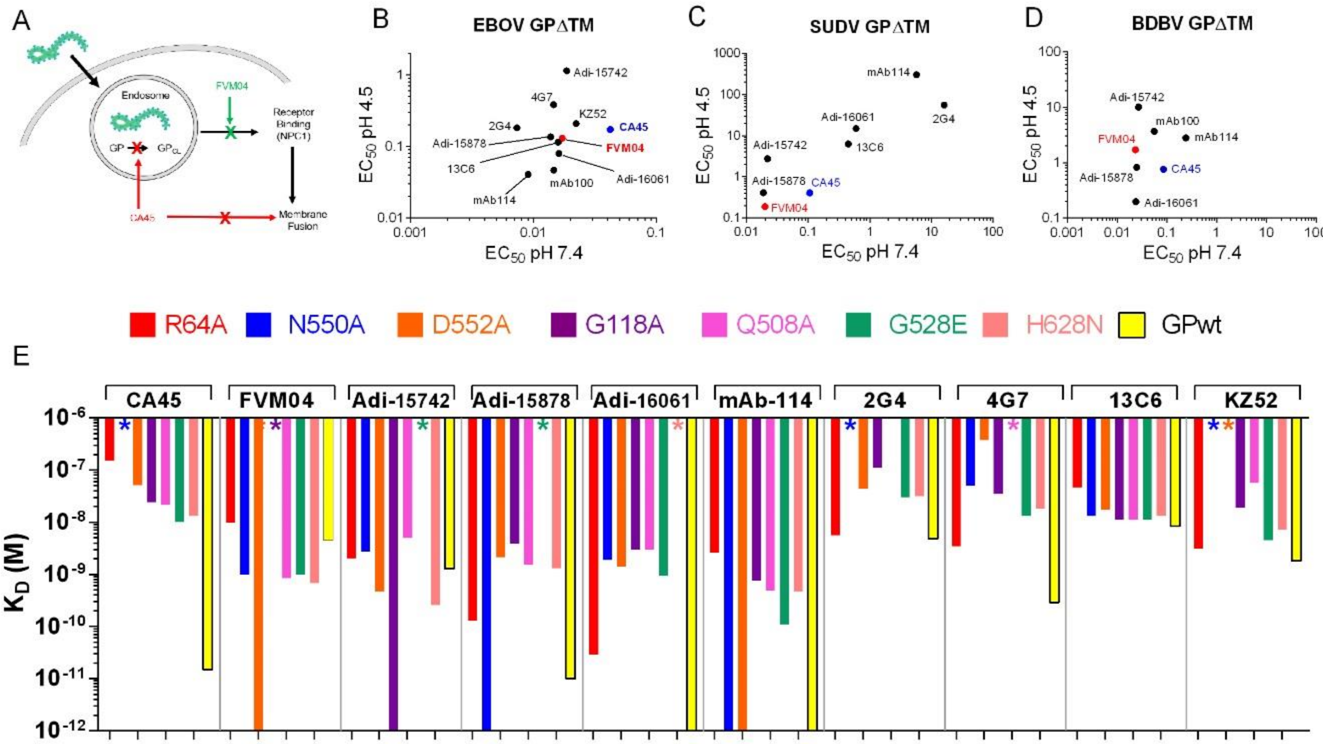

Supplementary Figure 1: Characteristics of FVM04 and CA45 in comparison with a panel of ebolavirus GP monoclonal antibodies. A) FVM04 and CA45 collectively block the three main steps of ebolavirus entry. B) Binding of the indicated antibodies to GP of three ebolavirus species at neutral and acidic pH. C) K_D values of the indicated antibodies for a panel of EBOV GP mutants. Base mutations: R64A, N550A, D552A, G528E, and Q508A. Stalk mutation: H628N. RBS mutation: G118A. Color coded asterisks indicate lack of binding of the respective antibody to the mutant with corresponding color

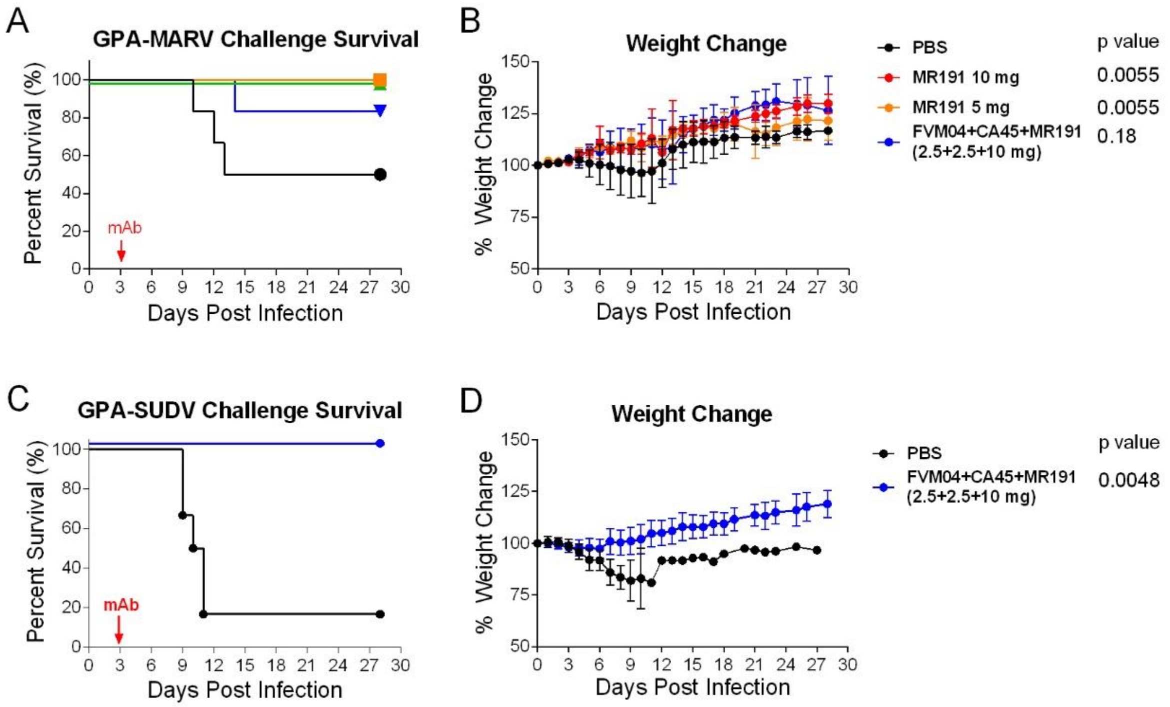

Supplementary Figure 2: Efficacy of PF cocktail (2.5 mg CA45+2.5 mg FVM04 + 10 mg MR191) in guinea pig model against GPA-MARV (A-B) when treated with 10 mg MR191 (p=0.055), 5 mg MR191 (p=0.055) or PF cocktail (p=0.18), or GPA-SUDV (C-D) when treated with PF cocktail (p=0.0048). All animals treated 3 dpi n=6 per group. Error bars represent the mean value of the 6 animals in each group ± Standard deviation (SD).

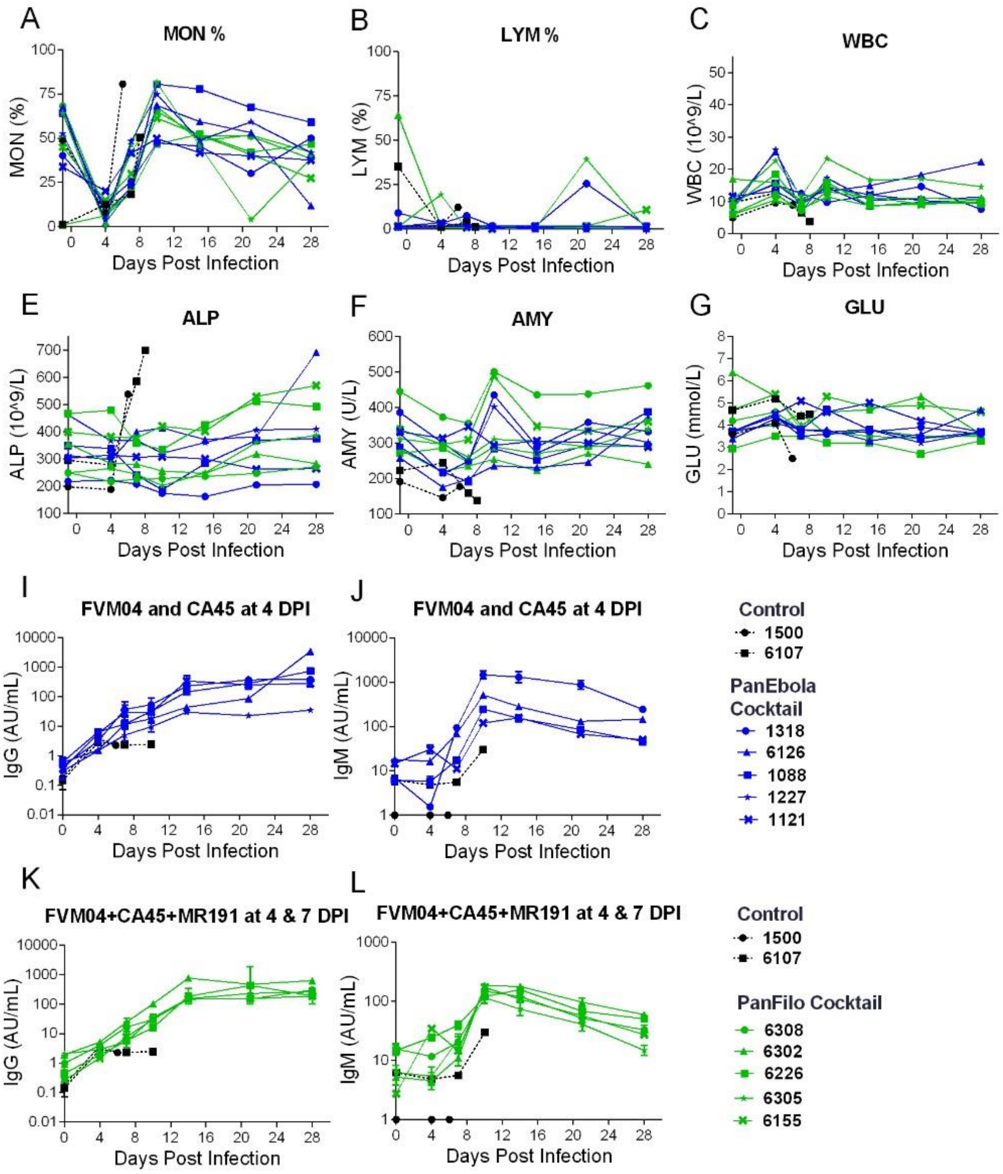

Supplementary Figure 3: Additional hematology (**A-D**), blood chemistry (**E-H**), and serology data (**I-L**) from the NHP EBOV efficacy study shown in main Fig. 2. MON: monocytes, LYM: lymphocytes, WBC: White blood cells, NEU: neutrophils, ALP: alkaline phosphatase, AMY: amylase, GLU: glucose, CRE: creatine.

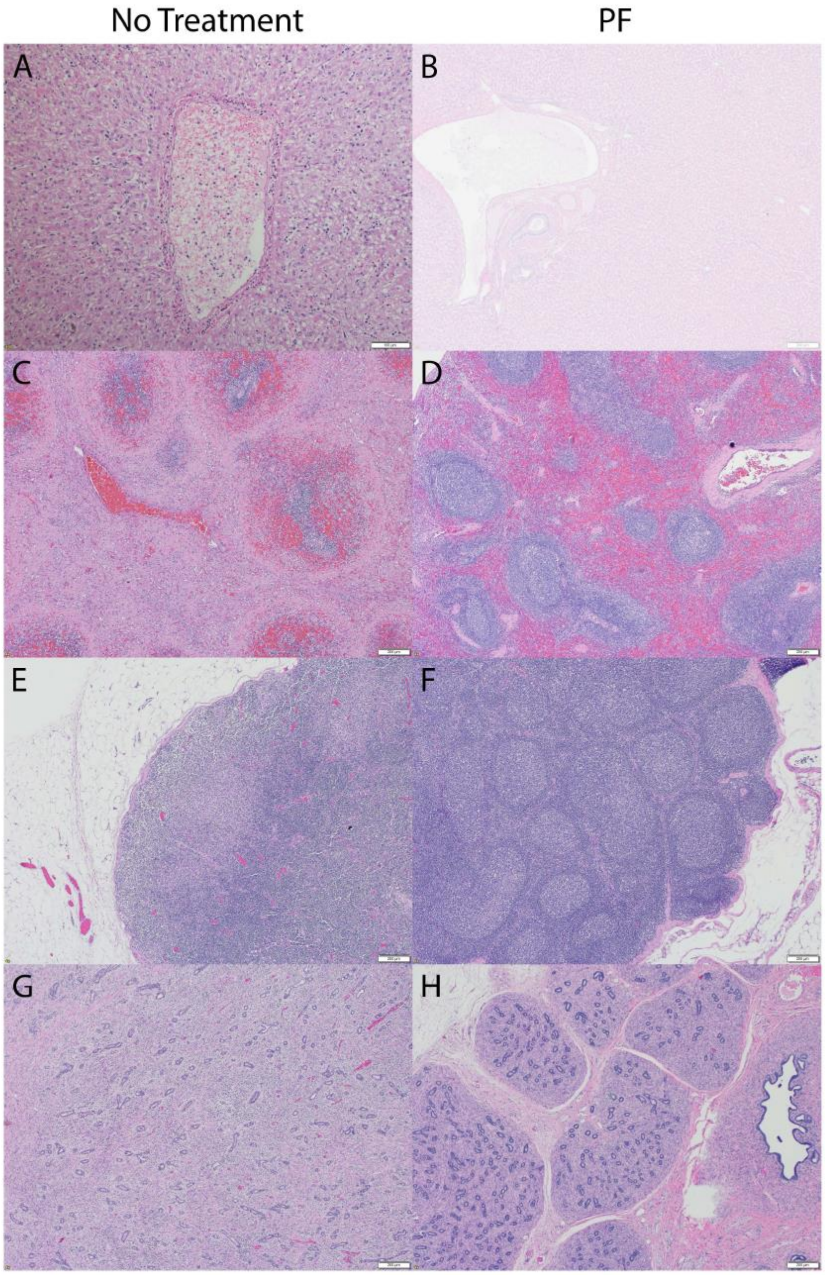

Supplementary Figure 4: Histologic features in control and PF-treated rhesus macaques inoculated intramuscularly with MARV. Tissues were collected following euthanasia at day 8 (control animal, A, C, E and G) or at day 27 (treated animal, B, D, F, and H). Sections were stained with hematoxylin and eosin (H&E). (A) Liver showing fibrinonecrotic vasculitis with hepatocellular degeneration and necrosis; (B) normal liver, no pathological signs of MVD. (C) Spleen with diffuse lymphoid depletion, perifollicular hemorrhage and fibrin deposition in the red pulp; (D) normal liver, no pathological signs of MVD; (E) inguinal lymph node showing moderate follicular lymphocytolysis; (F) inguinal lymph node with mild follicular lymphoid hyperplasia; (G) prostate demonstrating multifocal moderate histiocytic and lymphocytic prostatitis with necrosis; and (H) prostate, normal, immature, no signs of MVD. Scale Bar: 100 μm for A and 200 μm for B-H.

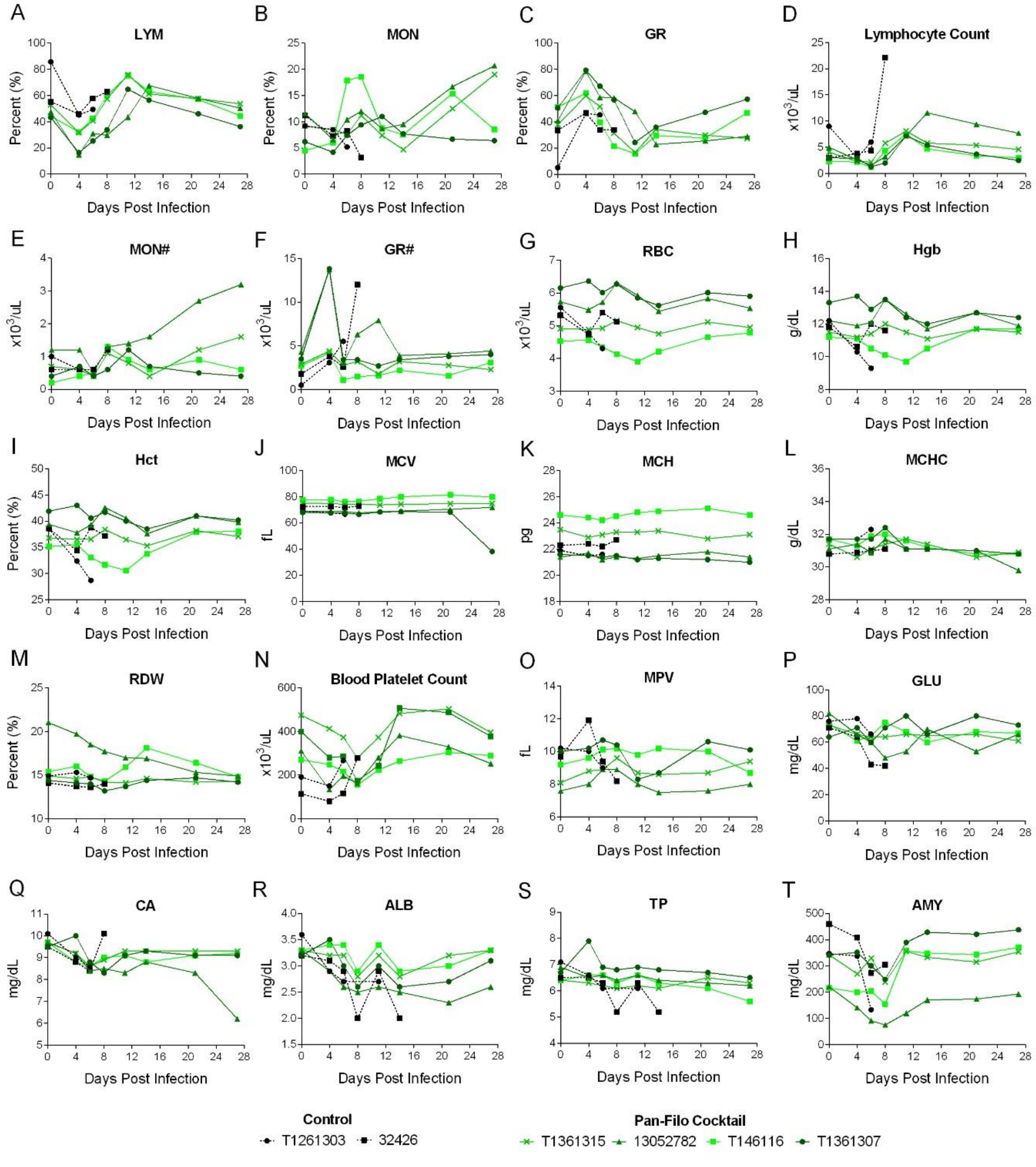

Supplementary Figure 5: Additional hematology (A-O) and blood chemistry (P-T) from the NHP MARV efficacy study shown in main Fig. 4. LYM: % lymphocytes, MON: % monocytes, GR: granulocytes, MON#: monocytes, GR#: granulocytes, RBC: red blood cells, Hgb: hemoglobin, Hct: hematocrit, MCV: mean corpuscular volume, MCH: mean

hemoglobin, MCHC: mean corpuscular hemoglobin concentration, RDW: red cell distribution width, PMV: mean blood platelet volume, GLU: glucose, CA: calcium, ALB: albumin, TP: total protein, AMY: amylase
