# Supplementary material for: Post-exposure immunotherapy for two ebolaviruses and Marburg virus in nonhuman primates
Source: Nat Commun. 2019 Jan 10;10:105. doi: 10.1038/s41467-018-08040-w (PMC6328579; doi:10.1038/s41467-018-08040-w)
Supplement: Supplementary file 3 — Reporting Summary [file 41467_2018_8040_MOESM3_ESM.pdf]

Corresponding author(s): M. Javad Aman
Xiangguo Qiu
John M. Dye

Reporting Summary

Nature Research wishes to improve the reproducibility of the work that we publish. This form provides structure for consistency and transparency in reporting. For further information on Nature Research policies, see Authors & Referees and the Editorial Policy Checklist.

Statistical parameters

When statistical analyses are reported, confirm that the following items are present in the relevant location (e.g. figure legend, table legend, main text, or Methods section).

n/a	Confirmed	
☐	☒	The exact sample size (n) for each experimental group/condition, given as a discrete number and unit of measurement
☒	☐	An indication of whether measurements were taken from distinct samples or whether the same sample was measured repeatedly
☐	☒	The statistical test(s) used AND whether they are one- or two-sided<br>*Only common tests should be described solely by name; describe more complex techniques in the Methods section.*
☒	☐	A description of all covariates tested
☒	☐	A description of any assumptions or corrections, such as tests of normality and adjustment for multiple comparisons
☐	☒	A full description of the statistics including central tendency (e.g. means) or other basic estimates (e.g. regression coefficient) AND variation (e.g. standard deviation) or associated estimates of uncertainty (e.g. confidence intervals)
☒	☐	For null hypothesis testing, the test statistic (e.g. F, t, r) with confidence intervals, effect sizes, degrees of freedom and P value noted<br>*Give P values as exact values whenever suitable.*
☒	☐	For Bayesian analysis, information on the choice of priors and Markov chain Monte Carlo settings
☒	☐	For hierarchical and complex designs, identification of the appropriate level for tests and full reporting of outcomes
☒	☐	Estimates of effect sizes (e.g. Cohen's d, Pearson's r), indicating how they were calculated
☐	☒	Clearly defined error bars<br>*State explicitly what error bars represent (e.g. SD, SE, CI)*

Our web collection on statistics for biologists may be useful.

Software and code

Policy information about availability of computer code

Data collection	BD FACSDiva Software to collect flow cytometry binding data<br>Applied Biosystems 7ABI 7500 Fast DX SDS Software v1.4 used for PCR machince
Data analysis	GraphPad Prism 6.0<br>Excel Office 365<br>FlowJo X was used to analyze the flow cytometry data<br>Pristima® Suite (Version 7.3.0 Build 23) used for pathology data management<br>SAS version 9.4 was used for the randomization

For manuscripts utilizing custom algorithms or software that are central to the research but not yet described in published literature, software must be made available to editors/reviewers upon request. We strongly encourage code deposition in a community repository (e.g. GitHub). See the Nature Research guidelines for submitting code & software for further information.

Data

Policy information about availability of data

All manuscripts must include a data availability statement. This statement should provide the following information, where applicable:

- Accession codes, unique identifiers, or web links for publicly available datasets
- A list of figures that have associated raw data
- A description of any restrictions on data availability

The data that support the findings of this study are available from the corresponding author upon reasonable request.

Field-specific reporting

Please select the best fit for your research. If you are not sure, read the appropriate sections before making your selection.

☒ Life sciences ☐ Behavioural & social sciences ☐ Ecological, evolutionary & environmental sciences

For a reference copy of the document with all sections, see nature.com/authors/policies/ReportingSummary-flat.pdf

Life sciences study design

All studies must disclose on these points even when the disclosure is negative.

Sample size	The reported studies are pilot studies. For Ebola and Marburg a sample size of 4 treated animals (4 used for Marburg, 5 for Ebola) provides adequate (>80%) power to detect a difference in response rates assuming at least 100% response in treated groups vs. 0% response in controls (2 experimental control + historical controls) at the 95% confidence level (1-tailed Fisher exact test). Because historical data using the identical Marburg or Ebola strains are available, the number of animals in the control groups was reduced to two. The use of two animals as controls relies solely on the assumption of a uniform, stable response of control animals over time. Uniform lethality has been observed experimentally in untreated rhesus NHPs at 1000 pfu IM for MARV-Angola (n ≥ 4) and for Ebola Makona (N>10). IACUC committees generally do not allow use of more than 2 controls for NHP filovirus models.<br>For Sudan study in Rhesus, this was the first time an immunotherapeutic was being tested in this model. As a pilot study we used n of 3 with 2 controls. However one of the controls survived, although this animal got very sick. Efficacy could be established based on lack of symptoms in two treated groups (total 6 animals). However due to partial lethality in the controls statistical significance in terms of protection could not be achieved.<br>For all guinea pig studies with n-6 were powered to detect significant survival difference above 50% between control and treatment.
Data exclusions	No data excluded
Replication	All ELISA assays were performed in intra-assay duplicates and data are presented as average. The error bars are too tight to show in the bar graphs.<br>For Sudan and Marburg animal studies: Plaque assays are averaged from two replicates, and PCR has three replicates.<br>For Ebola animal studies: TCID assays was performed with single point. PCR was performed in triplicate.<br>Flow cytometry raw data are mean fluorescence intensity averaged from 10,000 cells. Biolayer interferometry data are from single measurements over a concentration range.<br>Animal studies in guinea pigs are replicated in various designs. In general at least for one of the dose levels more than 3 experiments were performed.<br>NHP proof of concept studies were single experiments as replication of such experiments is not ethically acceptable.
Randomization	NHPs were randomized to groups stratified by sex. Randomization was performed using PROC PLAN in SAS Version 9.4
Blinding	Study directors were not blinded to the group allocations, however, the technical staff performing daily animal care, data collection, and making decision about euthanasia were blinded to group allocations until each study was completed.

Reporting for specific materials, systems and methods

Materials & experimental systems

n/a	Involved in the study
☒	☐ Unique biological materials
☐	☒ Antibodies
☐	☒ Eukaryotic cell lines
☒	☐ Palaeontology
☐	☒ Animals and other organisms
☒	☐ Human research participants

Methods

n/a	Involved in the study
☒	☐ ChIP-seq
☐	☒ Flow cytometry
☒	☐ MRI-based neuroimaging

Antibodies

Antibodies used

Anti Ebolavirus GP mAb FVM04; internally produced, sequence derived from patent WO2016069627
Anti Ebolavirus GP mAb CA45; internally produced, Genbank ID: KY859862 (IgH) and KY859863 (IgL)
Anti Marburgvirus GP mAb MR191: internally produced, Genbank ID: PDB6BP2
Anti Ebolavirus GP mAb ADI15742: internally produced, Genbank ID: KU602105 (IgH), KU602106 (IgL)
Anti Ebolavirus GP mAb ADI15878: internally produced, Genbank ID: KU602365 (IgH), KU602366 (IgL)
Anti Ebolavirus GP mAb ADI16061: internally produced, Genbank ID: KU602719 (IgH), KU602720 (IgL)
Anti Ebolavirus GP mAb mAb114: internally produced, Genbank ID: KU594603 (IgH), KU594604 (IgL)
Anti Ebolavirus GP mAb 2G4: internally produced, Genbank ID: PDB5KEL Anti Ebolavirus GP mAb 4G7: internally produced, Genbank ID: PDB5KEN Anti Ebolavirus GP mAb 13C6: IBT-Bioservices, Cat#0201-023, Lot#1708001
Anti Ebolavirus GP mAb KZ52: IBT-Bioservices, Cat#0260-001, Lot#1411001
Mouse monoclonal [SB108a] Anti-Rhesus monkey IgG heavy chain (HRP) preadsorbed: Source Abcam, Cat# ab99707, Lot#Gr315328-1
Goat anti-Monkey- IgM-HRP : Source Rockland, Cat#617-103-007
Goat-anti-Human IgG (H+L)-HRP : Source KPL, Cat#474-1006, Lot#10194727
Goat F(ab')2 Anti-Human IgG-PE: Source SouthernBiotech, Cat#2043-09, Lot#K0210-W121

Validation

The secondary antibody reacts with the heavy chain of human IgG. The specificity was validated by using VRC01 as a negative control, and various Ebola mAbs as positive control in the manuscript (Fig. 1A and 1B). All the Ebola antibodies produced internally were tested for reactivity to the corresponding GP antigens and tested for neutralization.

Eukaryotic cell lines

Policy information about cell lines

Cell line source(s)

ExpiCHO: was used for all the antibody production for NHP studies. Source: ThermoFisher Scientific, Cat# A29133.
HEK-293T cells: was used for some of the early antibody productions. Source ATCC, Cat#CRL-3216.
Vero C1008 (aka Vero E6): used for virus propagation, plaque assay, and TCID50. Source ATCC, Cat#CRL-1586.
S2 Drosophila cells (ExpreS2 Cells); ExpreS2ion Biotechnologies , Lot 13-1013

Authentication

The cell lines came from reputable vendors with certificate of analysis. No further authentication was performed. However, the behavior and morphology of the cells is routinely monitored.

Mycoplasma contamination

Integrated Biotherapeutics' 293T and Vero Cells were tested for mycoplasma in August 217 and the result was negative.
At USAMRIID all cell lines are tested monthly for mycoplasma. Cell lines used in this study tested negative.
PHA Canada's Vero-E6 cells were tested in July 2017 and were negative for mycoplasma.

Commonly misidentified lines
(See ICLAC register)

N/A

Animals and other organisms

Policy information about studies involving animals; ARRIVE guidelines recommended for reporting animal research

Laboratory animals

Ethical approval for animal studies were obtained from the IACUC Committees of USAMRIID and PHA Canada prior to start of the respective studies.
Hartley guinea pigs; 4-6 week old; female; vendor: Charles River (Wilmington, MA)
Rhesus macaques (Macaca mulatta) used in Sudan virus studies: 5 females, 3 males; age range: 5-6.6 years; weight range: 4.7-7.25 Kg
Rhesus macaques used in Ebola virus studies: 7 females and 5 males, age range: 3.75-4.75 years; weight range: 3.49-4.71 Kg
Rhesus macaques used in Marburg virus study: 3 males and 3 females; age range: 3.75-5.25 years; weight range: 4.45-5.55 Kg.

Wild animals

N/A

Field-collected samples

N/A

Flow Cytometry

Plots

Confirm that:

- [ ] The axis labels state the marker and fluorochrome used (e.g. CD4-FITC).
- [ ] The axis scales are clearly visible. Include numbers along axes only for bottom left plot of group (a 'group' is an analysis of identical markers).
- [ ] All plots are contour plots with outliers or pseudocolor plots.
- [ ] A numerical value for number of cells or percentage (with statistics) is provided.

Methodology

Sample preparation	HEK 293T cells were transfected with Makona GP variants using FuGENE HD following the manufacturer's instructions. Twenty-four hours later, cells were washed with PBS and detached by using 5 mM EDTA/PBS. The reaction was stopped by adding complete DMEM and gentle pipetting for single cell suspension. Cells were centrifuged at 1,200 rpm for 3 minutes and resuspended in cold PBS buffer with 2% FBS (FACS buffer) and then split equally into 96-well V-bottom plates for EBOV antibody staining. Staining was performed in a 100 µl cell suspension with 10 µg/ml primary EBOV antibody at 4°C. Thirty minutes later cells were washed with 200 µl FACS buffer for 3 times to remove the non-binding antibody. Secondary staining was then performed in a 100 µl suspension with 10 µg/ml goat anti-human IgG PE conjugate (SouthernBiotech, CAT# 2043-09) at 4°C in the dark. Thirty minutes later cells were washed 3 times with 200 µl FACS buffer and then fixed with fresh 2% PFA for flow cytometry analysis.
Instrument	FACSAria Special Order
Software	BD FACSDiva Software was used to collect the flow cytometry data. FlowJo X was used to analyze the flow cytometry data.
Cell population abundance	A total of 10,000 single cells of each staining was detected for binding of antibodies to Makona GP variants.
Gating strategy	(a) FSC-A vs. SSC-A plot with gate 1 to separate cell events from debris; (b) FSC-A and FCS-H plot with gate 2 to separate single cell events for cells of gate 1; (c) Histogram of PE fluorescence peak heights for cells of gate 2.

- [ ] Tick this box to confirm that a figure exemplifying the gating strategy is provided in the Supplementary Information.
